# The Impact of Family History of Allergy on Risk of Food Allergy: A Population-Based Study of Infants

**DOI:** 10.3390/ijerph10115364

**Published:** 2013-10-25

**Authors:** Jennifer J. Koplin, Katrina J. Allen, Lyle C. Gurrin, Rachel L. Peters, Adrian J. Lowe, Mimi L. K. Tang, Shyamali C. Dharmage, the HealthNuts Study Team

**Affiliations:** 1 Centre for Molecular, Environmental, Genetic and Analytic Epidemiology, University of Melbourne, Parkville, Victoria 3052, Australia; E-Mails: jennifer.koplin@mcri.edu.au (J.J.K.); lgurrin@unimelb.edu.au (L.C.G.); lowe.adrian@gmail.com (A.J.L.); s.dharmage@unimelb.edu.au (S.C.D.); 2 Murdoch Childrens Research Institute, Parkville, Victoria 3052, Australia; E-Mails: rachel.peters@mcri.edu.au (R.L.P.); mimi.tang@rch.org.au (M.L.K.T.); 3 Department of Allergy and Immunology, Royal Children’s Hospital, Parkville, Victoria 3052, Australia; 4 Department of Paediatrics, University of Melbourne, Parkville, Victoria, Australia

**Keywords:** food allergy, family history, genetics, heritability, siblings, maternal, paternal, egg allergy, peanut allergy

## Abstract

The apparent rapid increase in IgE-mediated food allergy and its implications are now widely recognized, but little is known about the relationship between family history (an indirect measure of genetic risk) and the risk of food allergy. In a population-based study of 5,276 one year old infants (HealthNuts), the prevalence of oral food challenge-confirmed food allergy was measured. Associations between family history of allergic disease and food allergy in infants were examined using multiple logistic regression. Food allergy was diagnosed in 534 infants. Compared to those with no family history of allergic disease, children meeting the current definition of “high risk” for allergic disease (one immediate family member with a history of any allergic disease) showed only a modest increase (OR 1.4, 95% CI 1.1–1.7) in food allergy, while having two or more allergic family members was more strongly predictive of food allergy in the child (OR 1.8, 95% CI 1.5–2.3). There were also differences in the associations between family history and egg and peanut allergy in the child. Re-defining “high risk” as two or more allergic family members may be more useful for identification of groups with a significantly increased risk of food allergy both clinically and within research studies.

## 1. Introduction

Asthma, eczema and allergic rhinitis have increased significantly in prevalence over the last 40 years [1]. The worldwide rise in these allergic diseases means that more people now may have one or more first degree relatives with an allergic disease. Interestingly, it is only in the recent past that food allergy prevalence appears to have increased substantially, a phenomenon which seems to have lagged behind the rise in other allergic diseases [1]. While it is acknowledged that this apparent increase in food allergy is likely related mainly to environmental risk factors given the short time frame involved, there is some evidence that heritability plays a role in food allergy. Family studies have shown that food sensitization and allergy are more common in those with a first degree relative with food allergy [2] and a twin study demonstrated higher concordance of peanut allergy in monozygotic compared with dizygotic twins [3]. 

On the other hand it is not clear whether the worldwide rise in other allergic diseases has played a role in the subsequent rise in food allergy. Currently it is widely accepted that children with a family history of ‘allergy’ in general are at increased risk of food allergy. If this is true, the increase in food allergy may be at least partly related to the rise in other allergies as this allergy epidemic has substantially increased the proportion with a family history of allergy. However, there is a scarcity of population data supporting this theory. This is because few large population-based studies have reported the risk of food allergy stratified by family history profile. Among those no studies have measured food allergy using the gold standard of challenge proven outcomes, which is required to accurately assess the risk of food allergy.

Understanding the relationship between family history of allergy and food allergy is important for: (1) clinical reasons (to tell families whether their children are at increased risk or general population risk of food allergy); (2) for identification of high risk groups that might be targeted for intervention for prevention of food allergy; and (3) understanding proxy measures of genetic susceptibility that can be used in gene-environment interaction studies. 

Using a population cohort we aimed to assess the impact of specific elements of family history of allergic disease on the risk of developing food allergy in the first year of life.

## 2. Methods

### 2.1. HealthNuts Study Methods

HealthNuts is a large-scale, population-based cohort study undertaken to assess the prevalence and risk factors for allergic disease in early childhood [4]. Briefly, we recruited 5,300 1 year old infants (73% participation rate) from population-based immunization clinics between September 2007 and August 2011. Parents completed a questionnaire which collected information about history of allergic disease in immediate family members as well as environmental exposures. Information on family history of allergic disease and history of food allergy and eczema in the infant was also collected for those who chose not to participate in the study. Demographics of study participants were generally representative of all infants born in Victoria, although participants were more likely to have a family history of allergic disease and infants were more likely to have eczema compared to those who declined to participate [4].

Family history of allergy was defined as reported asthma, eczema, allergic rhinitis or food allergy in a parent or sibling, using responses to the question “does anyone in your family suffer asthma, eczema, hay fever, food allergy (please specify food)”. Separate tick boxes were provided for each type of allergic disease, and for each family member (mother, father and siblings). 

All infants subsequently underwent a skin prick test (SPT) to egg, peanut and sesame and if positive, underwent an oral food challenge (OFC). Skin prick testing was repeated prior to OFC and food-specific IgE levels to egg, peanut and sesame were also measured. IgE-mediated food allergy (N = 534) was defined as a positive oral food challenge (hives, vomiting, angioedema or anaphylaxis (circulatory or respiratory involvement) within 2 h of a dose of the challenge food (egg, peanut or sesame)) in the context of IgE sensitisation to that food (SPT ≥ 2 mm or RAST ≥ 0.35) [5]. Infants with both evidence of IgE sensitisation and a recent history of reaction to the food in question (within the past 1 month for egg and 2 months for peanut and sesame) were classified as food allergic and did not undergo oral food challenge (n = 30).

Ethical approval was obtained from the Office for Children Human Research Ethics Committee (HREC) (ref. no. CDF/07/492), Department of Human Services HREC (ref. no. 10/07) and Royal Children’s Hospital HREC (ref. no. 27047).

### 2.2. Statistical Analysis

We initially used logistic regression to calculate odds of food allergy in the infant stratified according to the number of immediate family members with a history of any allergic disease (asthma, eczema, allergic rhinitis or food allergy). We also investigated whether the relationship between family history of allergy and food allergy in the participating infant differed according to any of the following: the infant’s gender; parents’ country of birth; infant history of eczema (reported diagnosis of eczema at any time in the first year of life); or having siblings. To do this, separate logistic regression models were fitted with and without interaction terms between each of these factors and family history of allergy. The models with and without interaction terms were then compared using the likelihood ratio test. 

To determine the prevalence of food allergy in infants according to family history of allergy, analyses were conducted separately for infants with and without siblings, since we have previously shown that infants with siblings are less likely to be food allergic and we wanted to predict the risk in both groups [6]. Prevalence was calculated as proportions with 95% CI calculated assuming a binomial distribution.

Next, multivariable logistic regression was used to examine the relationship between type of parent and sibling history of allergic disease and food allergy. A combined phenotype of asthma and allergic rhinitis (allergic rhinitis alone, asthma alone and asthma with allergic rhinitis) was used in the analysis [7]. Models were also adjusted for potential confounders, defined as those factors that changed the estimate by at least 10%. Potential confounders considered were presence of household pets (cats and dogs examined separately) and timing of introduction of egg into the infant diet. These factors were considered as potential confounders because they have been shown to be associated with risk of food allergy in this cohort [6,8] and because family history of allergy may cause parents to change behaviors with regard to keeping of pets and timing of egg introduction. All models were also adjusted for number of siblings. 

Any food allergy in this study was defined as the presence of challenge-proven egg, peanut or sesame allergy. Analyses are also presented separately for the outcomes of infant peanut and egg allergy, the two most common food allergies in this cohort. Sesame allergy was uncommon (n = 33) and was thus not examined as a separate outcome. We hypothesized that genetic susceptibility might differ for these two food allergies, which have a significantly different natural history. Analyses were performed using Stata (version 12) (StataCorp LP, College Station, TX, USA).

### 2.3. Sensitivity Analysis

We have previously reported that study participants were more likely to have a family history of allergic disease and infants were more likely to have eczema compared to those who declined to participate [9]. We therefore conducted a sensitivity analysis to determine whether the prevalence estimates were likely to be influenced by differences in participants and non-participants, using the propensity weighting method described by Little and Rubin [10]. Weights were used to adjust the estimated prevalence to reflect the distribution of risk factors among the combined sample of participants and nonparticipants. Weights were calculated as the inverse of the probability of participation. The probability was derived from information available for both participants and non-participants: sex of the child, socioeconomic status, family history of allergy, a previous diagnosis of eczema, the child’s number of siblings and whether the child was eating and tolerating peanut. 

## 3. Results

### 3.1. Prevalence of Family History of Allergy in the Population

Characteristics of the HealthNuts study population have been published previously [6,11]. Among infants participating in the HealthNuts study, 69% (95% CI 68.1, 70.6) had an immediate family member (parent or sibling) with a history of eczema, asthma, allergic rhinitis or food allergy. While allergic rhinitis was more commonly reported in parents, food allergies and eczema were more common among siblings (Table 1). The prevalence of asthma was similar among parents and siblings. 

**Table 1:** Prevalence of current allergic disease (%) among families of infants participating in the HealthNuts study (n = 5,276).

	Family member				
	Mother (n = 5,276)	Father (n = 5,276)	Brother (n = 1,556)	Sister (n = 1,475)	Any family member (n = 5,276)
Any allergy	44.2	37.9	37.5	35.7	69.4
Allergic rhinitis	30.7	27.2	9.6	6.9	50.0
Asthma	14.9	13.5	14.0	10.5	30.7
Eczema	14.1	7.6	24.4	24.3	30.5
Any food allergy	5.6	3.6	7.9	7.7	13.0
Peanut allergy	0.6	0.6	3.1	2.5	2.8
Egg allergy	0.3	0.3	2.4	2.1	1.8

### 3.2. Risk of Food Allergy according to Number of Immediate Family Members with a History of Allergic Disease

**Figure 1:**
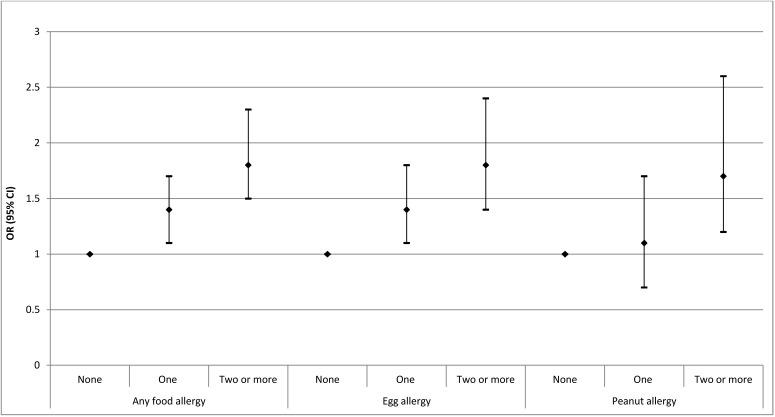
Crude odds ratio (with 95% CI) in for any food allergy according to number of immediate family members with a history of allergic disease.

Compared to having no allergic family members, having a single family member with a history of allergic disease increased the risk of food allergy in the child by 1.4 fold (Figure 1), while having two or more family members with a history of allergic disease increased the risk of food allergy by 1.8-fold. There was evidence that food allergy was more common among those with two or more allergic family members compared to only one allergic family member (*p* = 0.042). This pattern was consistent when egg allergy was examined as a separate outcome, but differed for peanut allergy. For peanut allergy, having a single family member with a history of allergic disease was not associated with an increased risk of peanut allergy (OR 1.1), while having two or more family members with a history of allergic disease significantly increased the risk of peanut allergy in the child (OR 1.7). There was modest evidence that peanut allergy was more common among those with two or more allergic family members compared to only one allergic family member (*p* = 0.065). 

There was no evidence that the relationship between number of family members with allergic disease and infant food allergy differed according to the infant’s gender or infant history of eczema (*p* > 0.05 for all interaction terms).

There was some evidence that the association between having two or more family members with allergic disease and infant food allergy was stronger among those with both parents born in East Asia compared to those with both parents born in Australia (*p* = 0.037 for interaction). OR for food allergy in infants with one allergic family member compared with no allergic family members: 1.9 (95% CI 1.3, 2.7) for infants with both parents born in Australia, 1.4 (95% CI 0.7, 2.9) for infants with both parents born in East Asia. OR for food allergy in infants with two allergic family members compared with no allergic family members: 2.2 (95% CI 1.5, 3.3) for infants with both parents born in Australia, 4.7 (95% CI 2.3, 9.5) for infants with both parents born in East Asia. 

There was no evidence that the relationship between parental history of allergy and infant food allergy differed according to the presence or absence of siblings (*p* = 0.11 for interaction). 

Among those with only one allergic parent, having a mother but not a father with allergic disease was associated with an increased risk of food allergy in the infant (OR 1.4, 95% CI 1.1–1.8, *p* = 0.004 and OR 1.2, 95% CI 0.9–1.2, *p* = 0.21 for maternal and paternal history respectively). However, the risk of infant food allergy was not significantly different between those with only a maternal and only a paternal allergic disease history (*p* = 0.21). 

### 3.3. Prevalence of Food Allergy according to Number of Immediate Family Members with Allergic Disease

Among those without any family history of allergy, food allergy was approximately twice as common in infants without siblings compared to those with siblings (Table 2; 10.2% *vs.* 5.6%; *p* = 0.001). However, food allergy was as common in infants with allergic siblings as in infants with no siblings, given an absence of parental allergic disease (10.3% *vs.* 9.6%, *p* = 0.77). 

Having a sibling with allergic disease almost doubled the risk of food allergy in the child compared with having no family history of allergy, even in the absence of parental history of allergy (9.6% *vs.* 5.6% in children with siblings, *p* = 0.025). Not surprisingly, the risk of food allergy was highest (20.0%) in those with both two allergic parents and one or more siblings with allergy. 

There were some differences between egg and peanut allergy. Unlike for egg allergy, the presence of siblings was not associated with peanut allergy among those without a family history of allergy (*p* = 0.65). The only groups with in an increased prevalence of peanut allergy compared to infants without a family history of allergy were those with two allergic parents and those with both an allergic sibling and at least one allergic parent. The prevalence estimates were similar after taking into account differences between participants and non-participants in the study (Table 3). 

### 3.4. Impact of Parental and Sibling Asthma, Allergic Rhinitis, Eczema and Food Allergy on Risk of Food Allergy in the Infant

In a multivariable model maternal history of eczema and asthma predicted egg allergy in the infant (Table 4). By contrast, only maternal and paternal history of asthma with allergic rhinitis predicted peanut allergy in the infant. Sibling history of food allergy was initially associated with an increased risk of egg allergy in the infant; however this association was no longer present after adjusting for timing of introduction of egg into the infant diet and presence of household pets.

Sibling history of both allergic rhinitis and asthma were associated with infant egg allergy in the adjusted model. No sibling allergic disease factors were significantly associated with peanut allergy in the infant. 

**Table 2:** Proportion of infants with food allergy, egg and peanut allergy stratified by number of allergic family members.

Does the infant have siblings?	Sibling history of allergy	Parent history of allergy	N	Any food allergy Prevalence (95% CI)	*p* value *	Egg allergy Prevalence (95% CI)	*p* value *	Peanut allergy Prevalence (95% CI)	*p* value *
No	-	None	858	10.3 (8.2, 12.3)	-	8.9 (7.0, 10.8)	-	2.6 (1.5, 3.6)	-
No	-	One parent	1,083	12.7 (10.8, 14.7)	0.090	11.8 (9.9, 13.8)	0.033	2.7 (1.7, 3.6)	0.87
No	-	Two parents	482	16.0 (12.7, 19.3)	0.002	13.9 (10.8, 17.0)	0.005	4.0 (2.3, 5.7)	0.14
Yes	No	None	648	5.6 (3.8, 7.3)	-	4.6 (3.0, 6.2)	-	2.2 (1.1, 3.4)	-
Yes	No	One parent	650	7.8 (5.8, 9.9)	0.10	6.4 (4.5, 8.2)	0.15	2.9 (1.6, 4.1)	0.47
Yes	No	Two parents	197	10.2 (5.9, 14.4)	0.025	8.9 (4.9, 12.8)	0.022	2.0 (0.04, 3.9)	0.81
Yes	Yes	None	270	9.6 (6.1, 13.2)	-	7.9 (4.7, 11.1)	-	2.5 (0.7, 4.3)	-
Yes	Yes	One parent	474	11.0 (8.1, 13.8)	0.57	7.9 (5.5, 10.3)	0.99	5.1 (3.1, 7.0)	0.090
Yes	Yes	Two parents	225	20.0 (14.7, 25.3)	0.001	19.1 (14.0, 24.3)	< 0.001	5.2 (2.3, 8.0)	0.12

* *p* values compare prevalence of food allergy among infants with no parental history of allergy with prevalence of food allergy among infants with one or two allergic parents, stratified by presence or absence of siblings and sibling history of allergy.

**Table 3:** Prevalence of food allergy stratified by family history of allergy, comparison of weighted and non-weighted results.

Does the infant have siblings?	Sibling history of allergy	Parent history of allergy	N	Prevalence of food allergy among responders (95% CI)	Prevalence of food allergy, using sampling weights (95% CI)
No	-	None	858	10.3 (8.2, 12.3)	9.6 (7.5, 11.6)
No	-	One parent	1,083	12.7 (10.8, 14.7)	12.1 (10.1, 14.1)
No	-	Two parents	482	16.0 (12.7, 19.3)	14.6 (11.3, 17.9)
Yes	No	None	689	5.6 (3.8, 7.3)	5.0 (3.3, 6.8)
Yes	No	One parent	684	7.8 (5.8, 9.9)	7.6 (5.4, 9.7)
Yes	No	Two parents	213	10.2 (5.9, 14.4)	9.0 (4.9, 13.1)
Yes	Yes	None	297	9.6 (6.1, 13.2)	10.0 (6.3, 13.8)
Yes	Yes	One parent	513	11.0 (8.1, 13.8)	10.9 (7.9, 13.8)
Yes	Yes	Two parents	251	20.0 (14.7, 25.3)	18.0 (12.7, 23.4)

**Table 4:** Relationship between type of family history of allergic disease and egg and peanut allergy in the child.

	Egg allergy				Peanut allergy			
	Crude multivariable model *		Adjusted for confounders ^†^		Crude multivariable model *		Adjusted for confounders ^†^	
	OR (95% CI)	*p*	OR (95% CI)	*p*	OR (95% CI)	*p*	OR (95% CI)	*p*
*Maternal allergy*								
Food Allergy (n = 297)	0.76 (0.50, 1.16)	0.21	0.65 (0.41, 1.04)	0.075	0.46 (0.18, 1.14)	0.092	0.35 (0.12, 0.97)	0.044
Eczema (n = 742)	1.65 (1.28, 2.12)	<0.001	1.57 (1.20, 2.04)	0.001	1.31 (0.85, 2.03)	0.22	1.27 (0.81, 2.00)	0.30
Allergic rhinitis alone (n = 1,167)	1.22 (0.97, 1.55)	0.088	1.27 (0.99, 1.63)	0.052	1.18 (0.79, 1.77)	0.42	1.26 (0.83, 1.92)	0.28
Asthma alone (n = 377)	1.41 (0.98, 2.03)	0.066	1.54 (1.05, 2.26)	0.027	1.33 (0.71, 2.51)	0.37	1.48 (0.76, 2.86)	0.25
Allergic rhinitis + asthma (n = 450)	1.23 (0.87, 1.73)	0.24	1.30 (0.91, 1.86)	0.15	1.97 (1.18, 3.28)	0.009	2.35 (1.39, 3.96)	0.001
*Paternal allergy*								
Food Allergy (n = 192)	0.89 (0.53, 1.50)	0.67	0.77 (0.44, 1.36)	0.37	0.87 (0.35, 2.18)	0.77	0.72 (0.26, 2.01)	0.54
Eczema (n = 401)	1.52 (1.10, 2.09)	0.011	1.35 (0.96, 1.90)	0.086	0.82 (0.43, 1.56)	0.55	0.76 (0.39, 1.49)	0.42
Allergic rhinitis alone (n = 1,047)	1.20 (0.94, 1.52)	0.14	1.21 (0.94, 1.56)	0.13	1.28 (0.86, 1.91)	0.23	1.28 (0.85, 1.93)	0.25
Asthma alone (n = 326)	1.09 (0.73, 1.63)	0.67	1.21 (0.80, 1.82)	0.37	0.71 (0.31, 1.64)	0.42	0.77 (0.33, 1.79)	0.54
Allergic rhinitis + asthma (n = 388)	1.21 (0.85, 1.72)	0.29	1.14 (0.79, 1.67)	0.48	1.90 (1.13, 3.21)	0.016	1.77 (1.01, 3.09)	0.045
*Siblings*								
Food Allergy (n = 240)	1.68 (1.08, 2.62)	0.023	1.25 (0.77, 2.05)	0.36	0.97 (0.45, 2.10)	0.94	0.78 (0.34, 1.79)	0.56
Eczema (n = 740)	1.31 (0.94, 1.83)	0.11	1.26 (0.89, 1.78)	0.20	1.34 (0.81, 2.19)	0.25	1.23 (0.73, 2.06)	0.44
Allergic rhinitis alone (n = 157)	1.93 (1.15, 3.24)	0.013	2.45 (1.45, 4.17)	0.001	1.42 (0.62, 3.23)	0.41	1.65 (0.71, 3.82)	0.24
Asthma alone (n = 302)	1.44 (0.94, 2.22)	0.095	1.56 (1.00, 2.43)	0.051	1.52 (0.82, 2.81)	0.19	1.59 (0.85, 3.00)	0.15
Allergic rhinitis + asthma (n = 78)	1.34 (0.61, 2.97)	0.47	1.66 (0.74, 3.71)	0.21	0.32 (0.04, 2.43)	0.27	0.35 (0.05, 2.65)	0.31

* All parental and sibling allergic disease variables were included in the model simultaneously and are therefore adjusted for each other. The models were also adjusted for number of siblings; ^†^ Confounders were timing of introduction of egg and pet ownership.

## 4. Discussion

In this large population-based study with a high participation rate we found almost 70% of the participants had a family history of allergy. However, children who met the commonly used definition of “high risk” for allergy (one immediate family member with a history of allergic disease) showed only modest increased risk of food allergy compared to those with no family history of allergy, while having two or more family members with a history of allergy was more strongly predictive of food allergy in the child. Among infants with siblings, both parental and sibling history of allergic disease were important predictors of food allergy in the infant, while among infants without siblings having two allergic parents predicted food allergy in the infant. Overall maternal history of allergic disease appeared to be more strongly associated with infant food allergy compared with paternal history of allergic disease.

There were differences in the associations between family history of allergic disease and egg and peanut allergy in the child, with maternal history of eczema and asthma and sibling history of allergic rhinitis predicting egg allergy in infants while maternal and paternal history of asthma with allergic rhinitis were the only predictors of infant peanut allergy. Although sibling history of food allergy was initially associated with risk of egg allergy in the child, this association was no longer present after controlling for timing of introduction of egg. This suggests that infants with food allergic siblings were at increased risk of egg allergy because of delayed introduction of egg rather than genetic factors. Changes in behavior may also explain the apparent protective effect of maternal history of food allergy on peanut allergy in the offspring. Interestingly, family history of asthma and allergic rhinitis predicted food allergy in the child independent of family history of food allergy. In fact history of these allergic diseases seemed more important in predicting childhood food allergy than history of food allergy in family members, although this needs to be considered in the context of potential misclassification of food allergy status in family members and lack of statistical power to take parental food allergy into account given the small prevalence of food allergy among parents. 

The association between family history of allergy and infant food allergy differed between infants of Australian-born and Asian-born parents. In this study we have observed lower rates of reported allergy among parents born in East Asia [12], but conversely higher rates of food allergy among their infants. This may indicate interactions between genes and the Australian environment and suggests that environmental factors associated with increased allergy work differentially on different genotypes. 

HealthNuts is the largest single-center study of infant food allergy to use oral food challenges to diagnose food allergy. It is uniquely suited to examine the relationship between family history of allergic disease and infant food allergy because of its population-based sampling strategy and the use of non-participant questionnaires to collect information on family history of allergy and surrogate measures of likely infant food allergy for all those who chose not to participate in the study. This allows an assessment of the impact of selection bias on the association between family history of food allergy and infant food allergy. Because of the high participation rate in this study, prevalence estimates changed little after taking into account differences between participants and non-participants, thus our results can be applied to the general population from which this study sample was derived. 

The main limitation of this study is the use of parent-reported family history of allergic disease to define family history of allergy, with information gathered from questionnaires mostly completed by the child’s mother (data not shown). As such, non-differential misclassification of family history could have contributed to the observed null associations. This may also explain the relatively lower magnitude of the association for paternal history of allergic disease, which was predominantly reported by mothers and therefore might be less accurate. However, the same definition is often used in clinical settings and epidemiological studies to identify those thought to be at high risk of food allergy, thus the information that this study provides about the relationship between self-reported family allergic disease and infant food allergy is valuable both for research and clinical practice. 

Additionally, this study focused mainly on three food allergies in childhood, namely egg, peanut and sesame allergy. Skin prick testing was also performed for cow’s milk allergy and shellfish; however food challenges were not performed for these foods. Overall food allergy as examined as an outcome therefore mainly reflects egg and peanut allergy, with egg allergy being the most common food allergy in this cohort. Milk allergy is also a common food allergy in infancy, however in this cohort IgE-mediated sensitization to milk at 12 months of age was rare, particularly among infants without any other food allergies (SPT ≥ 2 mm prevalence in these infants was 0.7%; data not shown). This suggests that we are unlikely to have misclassified a large number of milk allergic infants as not having an IgE-mediated food allergy. 

The relationship between family history of allergy and childhood food allergy has previously been considered in only a few population-based studies. Consistent with our findings, one previous birth cohort study in Korea (n = 1,177) reported that maternal atopic dermatitis was associated with an increased risk of parent-reported food allergy in the first year of life [13]. Egg allergy was the most common food allergy reported, but food challenges were not performed to confirm allergy status. A second small cohort study of 452 infants in Thailand reported that family history of allergy was associated with parent-reported food allergy, but was unable to assess associations with challenge-confirmed food allergy as only five infants had challenge-confirmed food allergy [14]. A study in Finland found that the cumulative incidence of any positive food allergy test by age four years (including SPT or sIgE testing) was threefold higher if both biologic parents had an allergic manifestation and twofold higher if one parent had an allergy than if both parents were free of allergies [15]. 

Two studies have examined challenge-proven food allergy to individual foods. In the Avon Longitudinal Study of Parents and Children, which included 23 children with challenge-confirmed peanut allergy, maternal atopy was associated with peanut allergy in an unadjusted model, but the association was no longer significant after adjustment for environmental factors, possibly due to limited statistical power [16]. A recent case-control study involving case and control infants selected from a population-based study of cow’s milk allergy found that maternal but not paternal self-reported atopic disease was more common among infants with persistent IgE-mediated cow’s milk allergy, although there was no evidence of an association between objectively measured atopy (by SPT) in parents and milk allergy in the infant [17]. The finding that maternal self-reported atopic disease was more strongly associated with infant food allergy compared with paternal history is consistent with our findings. 

These findings have several implications for future studies. Firstly, restricting the sampling frame for intervention studies to “high risk infants”, defined on the basis of having one or more allergic family members, does not greatly increase the prevalence of food allergy as an outcome, particularly for peanut allergy. Studies targeting high risk infants may need to restrict to those with two or more allergic family members, particularly given the high prevalence of reported family history of allergy now present in the general population. However, the other side of this is that the high prevalence of family history of allergy means that results of studies enriched for a single family history will be applicable across the majority of the population. Additionally, two or more allergic family members may be a better proxy measure of genetic susceptibility that can be used in gene-environment interaction studies. 

Secondly, these results highlight some potential differences in the genetics of egg and peanut allergy. Sibling history of allergic disease was more strongly associated with infant egg allergy than with infant peanut allergy while maternal allergic asthma was the only independent predictor of peanut allergy. This may suggest that egg allergy is more strongly influenced by shared environment, while genetic factors might be more important in peanut allergy. Peanut and egg allergy vary considerably in terms of their prognosis, with most children outgrowing egg allergy while peanut allergy is more likely to be lifelong [18]. There is also evidence from our own work within the HealthNuts cohort that there are differences between infantile egg and peanut allergy at the molecular level [19]. Some of the differences between egg and peanut allergy observed here may be attributable to the lower sample size of peanut allergic infants, however this area warrants further investigation.

Finally, sibling history of allergy, which is only rarely reported in food allergy studies, should be taken into account in future studies. Having a sibling history of allergy is associated with an increased risk of food allergy in infants, even after taking into account parental history. Our results also suggest that sibling history of allergy may lead parents to change the environment in which subsequent infants are raised, providing an important potential source of confounding in observational studies investigating environmental risk factors for food allergy. 

## 5. Conclusions

Re-defining “high risk” of food allergy as infants with two or more allergic family members may be more useful for identification of groups with a significantly increased risk of food allergy both clinically and within research studies. 
